# The pathways between female garment workers’ experience of violence and development of depressive symptoms

**DOI:** 10.1371/journal.pone.0207485

**Published:** 2018-11-15

**Authors:** Kausar Parvin, Mahfuz Al Mamun, Andrew Gibbs, Rachel Jewkes, Ruchira Tabassum Naved

**Affiliations:** 1 Health Systems and Population Studies Division, icddr,b, Dhaka, Bangladesh; 2 Gender and Health Research Unit, South African Medical Research Council, Pretoria, South Africa; 3 School of Public Health, University of the Witwatersrand, Johannesburg, South Africa; Columbia University, KAZAKHSTAN

## Abstract

**Background:**

The prevalence of intimate partner violence (IPV) is high (54%) in Bangladesh. Moreover, female garment workers report higher rates of IPV and are also vulnerable to workplace violence (WPV). Experience of violence puts women at increased risk of developing depressive symptoms, which are related with low self-esteem, lower life satisfaction and lower productivity. To our knowledge, there has been no previous research on depression among female garment workers and its connections to IPV and WPV in Bangladesh. This paper aims to address this gap by studying the relationship of IPV, WPV and depression among female garment workers.

**Methods:**

The data for this paper comes from a cross-sectional survey of female garment workers (n = 800) conducted as baseline survey of a quasi-experimental study known as HERrespect. Survey data were collected during September-December, 2016 among randomly selected female garment workers from eight garment factories in and around Dhaka city. Structural equation modelling was conducted to explore the relationship among IPV, WPV and depression.

**Results:**

The findings show high rates of any IPV (69%); WPV (73%, experienced or witnessed) and depressive symptomatology (40%) among female garment workers. The analysis of pathways shows that IPV impacts a woman’s experience of WPV and work related stress leading to the development of depression; while WPV had direct and mediated pathways to depression. Experience of controlling by their husband leads to WPV and increased work related stress, and thus leads to depression. It also reveals that a worker’s ability to mobilize resources in emergency, however, increased self-esteem and reduced work related stress.

**Conclusion:**

This study shows the pathways through which experience of IPV and WPV lead to development of depressive symptoms among female garment workers. The link between women’s ability to mobilize resources with self-esteem and work related stress indicates the need for socio-economic empowerment of women and may suggest that combined intervention to address IPV and women’s empowerment could be successful in dealing with WPV and mental health.

## Introduction

Violence against women (VAW) is a well-recognized and serious social, human rights and public health problem [1,2]. It incurs substantial costs to nations [3]. Though there are numerous perpetrators of VAW, intimate partners remain the most common perpetrators [4]. Intimate partner violence (IPV) was ranked 31^st^ among the risk factors for the loss of global disability-adjusted life years in 2016[5]. There is considerable regional variation in its prevalence. Globally between 15 and 71% of ever-partnered women experience physical and/or sexual IPV in their lifetime [1]. In South Asia, the estimated prevalence of lifetime physical and/or sexual IPV is 42% [6].

Overall, the level of IPV in Bangladesh is high with 54% of ever-married women reporting lifetime physical and/or sexual IPV and 27% reporting such violence during the last 12 months [7], even though the national legislation regarding VAW in Bangladesh is in place [8,9]. According to available statistics, women who earn an income report more IPV compared to their non-income earning peers, as do poorer women. Research with female garment workers suggests that there may be a higher level of IPV (53% in the past 12 months) compared to the overall income earning female population (33% in the past 12 months) [7,10]. The literature from Bangladesh and elsewhere shows that women’s experiences of IPV are associated with a wide range of physical and mental health consequences for women [11,12]. IPV is also known to affect women’s work and productivity [13,14].

Previous studies conducted amongst garment workers in Bangladesh indicate that female garment workers also experience high levels of IPV, and workplace violence (WPV). In workplaces workers are subject to verbal, physical and sexual abuse [15–17]. Studies suggest that around 60% of female garment workers report verbal or physical abuse [16] in the workplace. Sexual harassment in the workplace is also common [16,17]. There is evidence of adverse consequences of WPV on workers’ physical and mental health [18,19] and job performence [20]. Thus, it would be expected that female garment workers experience greater vulnerability to mental health problems being exposed to both IPV and WPV.

Depression is one of the most common adverse mental health outcomes of VAW [12,21]. Women who experience IPV are twice as likely to develop depressive symptoms, than those who don’t experience IPV [4,12]. Similarly, WPV has been shown to increase depressive symptoms [22]. Depressive disorders are ranked as the single largest contributor to non-fatal health loss [23]and is a precursor of other mental and chronic health problems [24,25]. Moreover, depression is related to low self-esteem, lower life satisfaction and lower productivity [26–28]. Despite the adverse impact of depression, it has not been studied rigorously; the estimated prevalence of depression among adults in Bangladesh is 4. 1% [23], and not a priority area for health care delivery systems as mental health problems are not considered health problems [29]. However, considering the needs of VAW survivors, the Government has implemented the Multi-Sectoral Programme on Violence Against Women under the Ministry of Women and Children Affairs, which is meant to provide medical, legal, psychological support and rehabilitation to the survivors of VAW. Unfortunately mental health services are inadequate [30].

To our knowledge there has been no previous research on depression among female garment workers and its connections to IPV and WPV, in the context of Bangladesh. Based on the literature cited above, we hypothesised that women who experience IPV and WPV are more likely to report depressive symptoms, and further that IPV and WPV are interrelated and their combined effect exacerbates workers’ mental ill-health. To investigate this we present an analysis of data collected in research with female garment workers in factories in Bangladesh and use structural equation modelling to understand the pathways through which IPV and WPV impact the development of depressive symptomatology.

## Methods

### Design

This study uses baseline (cross-sectional) data of the HERrespect trial, an evaluation of an intervention aiming to reduce IPV and WPV against female garment workers in Bangladesh. The details of the methodology are described elsewhere [31]. Briefly, the HERrespect trial employs a quasi-experimental design, involving four intervention and four control factories in and around Dhaka city. One out of eight factories was from Export Processing Zones (EPZ). The factories were recruited through buyers in the garment industry for inclusion in the research.

The total sample size was 800 female garment workers (100 from each factory). A worker was eligible to be a study participant if she was currently married and living with her husband, had been working in the current factory for at least 12 months and willing to participate in the study. The final sample was randomly selected from eligible female workers list obtained from the factories. Baseline data were collected between September and December 2016 using face-to-face interviews in private in a location convenient for participants outside the factory, with data captured using Personalized Digital Assistants (PDAs).

### Measurement

#### Outcome

Depression in the past week was measured using Center for Epidemiologic Studies Depression (CES-D) scale. It has been used in many different settings (including Bangladesh) with high reliability and validity [32]. It is a 20-item 4-point ordinal scale. The responses are recorded as: rarely or none of the time (less than 1 day); some or a little of the time (1–2 days); occasionally or a moderate amount of the time (3–4 days); and most or all of the times (5–7 days). A summative score was derived based on the responses. We followed the cut-off point recommended by the scale to define presence of depressive symptomatology, which is 16 [32]. The scale shows high reliability among this population (Cronbach’s Alpha = 0. 87). The continuous score was used in the path model as the outcome variable where higher scores represents higher depression.

#### Covariates

Intimate partner violence (i. e. physical, sexual, emotional, economic violence) was measured using a set of questions based on the WHO violence against women instrument [33]. These instruments are designed to minimize reporting biases that arise from subjective perceptions of abuse by asking only about specific behaviours perpetrated by a male partner (e. g., “Has your husband ever slapped you or threw something at you that could hurt you?). Questions captured behaviours that reflect minor and severe physical violence (e. g., slapped, pushed, hit with a fist, kicked, dragged, choked, burned, threatened with a weapon); sexual violence (e. g., forced sexual intercourse or other sexual acts); emotional violence (e. g., insulted, humiliated); economic violence (e. g., prohibited from earning, thrown out of house) during the last 12 months. We created a dummy variable “any IPV” if a worker had affirmative response to any item of the four forms(physical, sexual, economic and emotional) of IPV in the last 12 months.

A modified version of sexual relationship power scale [34] was used to measure controlling behaviours by the garment worker’s husband. Items included: “He (husband) has more to say than you do about important decisions that affect both of you”; “He tells you who you can spend time with” etc. Responses were measured using 4-point Likert scale. A summative score was then used to assess control, with higher scores indicating more controlling behaviours by her husband. Cronbach’s Alpha for the scale was 0. 66.

An adapted peer victimisation scale [35] was used to measure workplace violence (WPV). The adaptation was made to measure ‘experiencing or witnessing’ victimisation as this was deemed to be less sensitive with factory management (who had to approve the survey prior to use) than just actual reported victimisation. The adaptation of questions drew heavily on the findings of formative research on the types of violence, which occur in the workplace [15]. The questions have 4-category scaled response options (never, once, few times, many times) and a typical item was ‘How often within the past 4 weeks have you experienced or witnessed a manager call an operator or helper names?” A dummy variable “any WPV” was then generated to report any workplace violence considering the items used. We used the continuous summative score of WPV (where a higher score was indicative of a higher level of WPV) in the path model. Cronbach’s Alpha was 0. 83 for the scale.

The Rosenberg Self-Esteem Scale (RSE) was used to measure self-esteem [36]. It is a 10-item scale that measures global self-worth by measuring both positive and negative feelings about self. The scale is believed to be uni-dimensional. All items are answered using a 4-point Likert scale format ranging from strongly agree to strongly disagree. A summative score was derived where higher score means higher self-esteem and the continuous score was included in the path model. For the self-esteem scale the Cronbach’s Alpha was 0. 76.

Work related stress was measured using three statements related to work and income, specifically, “you are frequently stressed or depressed because of not having enough income”; “you are frequently stressed or depressed because you are not proud of what you do to get money”; and “you are frequently stressed or depressed because you want or have to help your family with money”. Responses for all items were measured using a 4-point Likert scale ranging from strongly disagree to strongly agree. A continuous summative score, with higher scores indicating higher stress, was used in the path model.

To measure life satisfaction we used four items from of the Diener [37] scale on life satisfaction. The items are “In most ways your life is close to your ideal”; “The conditions of your life are excellent”; “You are satisfied with your life”; and “So far you have got the important things you wanted in life”. A 5-pointLikert scale was offered with options ranging from strongly disagree to strongly agree was used to record respondent’s answer. A continuous score was used, where higher scores reflect higher life satisfaction. Cronbach’s Alpha for this scale was 0. 88.

Disability, as a measure of worker’s health status, was defined by asking questions adapted from the Washington Group Short Set of Questions on Disability [38]. There are six questions related to having difficulties in doing certain activities such as difficulties in hearing, in walking or climbing steps, in washing all over or dressing, in remembering or concentrating etc. Responses ranged from ‘cannot do at all’ to ‘no difficulties’. A summative score was used in the path model, where higher score means less disability.

Ability to mobilize resources was determined by asking a single question “If you had an emergency at home and needed 50,000 Tk, (USD 599) how easy would you say it would be to find the money?”. The response options are very difficult, somewhat difficult, fairly easy and very easy.

In addition, workers age, level of education, age at marriage, number of children, NGO membership and income were also included in the analysis.

### Statistical analyses

All analyses were performed using STATA version 13. Descriptive analyses were performed to describe the characteristics of the sample, to state the rates of the outcome variable and the covariates. Range and standard deviations were reported where applicable. Bivariate analyses were also performed to describe associations between the main variable (depressive symptomology) and covariates (Chi-square and F-test tests for categorical variables; t-tests for continuous variables). Multivariate logistic regression analysis was performed to determine the factors associated with depression. For all statistical tests the p-value was set at 0. 05.

**Fig 1:**
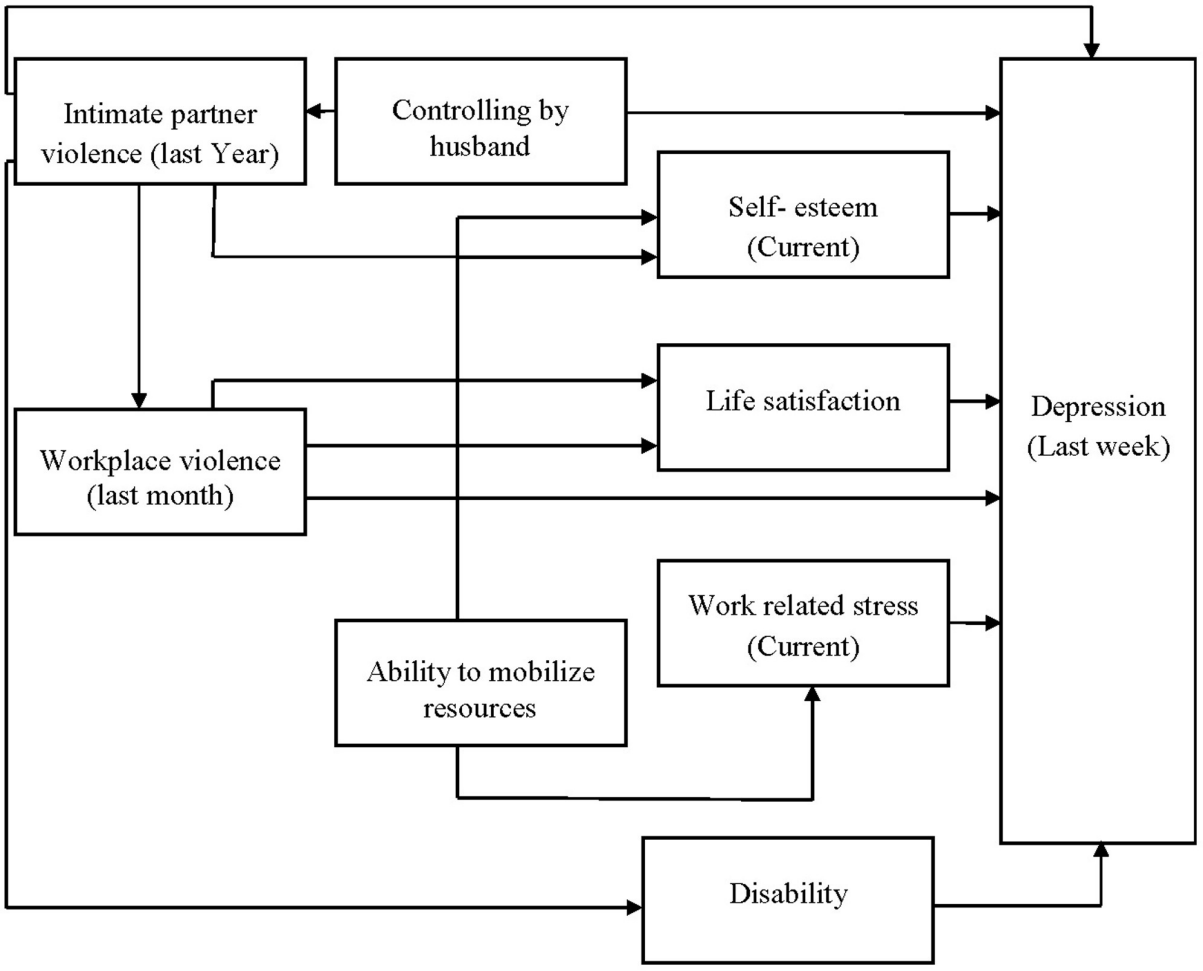
Conceptual framework of individual experience of intimate partner violence, workplace violence and development of depression.

Structural Equation Modelling (SEM) was conducted to assess the inter-relationship between variables and to explore the path between IPV, WPV and depression. The variables included in the model were based on bivariate analyses, regression analysis, theory and conceptual framework (Fig 1). We fitted a path model using maximum likelihood missing values (mlmv) estimation to model all available data. Insignificant (p value >0. 05) paths from the exogenous variables to the outcome variable and mediation paths were removed from the model using backward elimination. With each path removed we tested the model again. The final model was built based on theory and statistically meaningful modifications. To asses model fit of the observed data, we used the comparative fit index (CFI >0. 95); Tucker-Lewis Index (TLI >0. 95) [39] and root mean square error of approximation (RMSEA ≤0. 05) [40].

### Ethics

The study received approval from Institutional Review Board of icddr,b (PR#16036)and the South African Medical Research Council (SAMRC) ethics committee (PR# EC013-5/2016). The study followed the WHO guidelines on researching VAW [41]. All the interviews were conducted only upon receiving verbal consent from the participants considering low readability and high concerns about confidentiality in Bangladesh. Participants were free to stop the interview at any time and refuse to answer any question that they did not want to. No identifying information was recorded in the questionnaire and kept in a separate file to which only the researchers have access. The research findings will be presented in sufficiently aggregated form to ensure that no participating worker can be identified.

## Results

The female garment workers were 27 years old on average, with a mean of five years of education and a monthly average income of BDT 8,505(USD 109). One-third (34%) of workers reported physical IPV, 43% sexual IPV, half emotional IPV, and a third (35%) of them reported economic IPV during the last 12 months. Just under three quarters of the workers (69%) reported any IPV in the past year. Three in every four workers (74%) experienced or witnessed WPV. Around 40% of the workers reported potentially clinically relevant depressive symptomatology (Table 1).

**Table 1:** Descriptive statistics of socio-demographic characteristics and life experiences of female garment workers, n = 800.

Variable	Statistics
Mean age in years (range, SD)	27. 40 (17–57, 5. 70)
Mean education in years (range, SD)	5 (0–15, 3. 37)
Average current earning per month (SD)	8,505 (5000–12500, 1353)
Presence of depressive symptomatology (%)	39. 75
Any physical IPV (last 12 months) (%)	34. 38
Any sexual IPV (last 12 months) (%)	42. 75
Any emotional IPV (last 12 months) (%)	49. 60
Any economic IPV (last 12 months) (%)	35. 13
Any IPV (last 12 months) (%)	69. 00
Any workplace violence (last month) (%)	73. 50
Mean score Controlling by husband (range, SD)	3. 06 (0–9, 1. 76)
Mean score Disability(range, SD)	23. 03 (16–24, 1. 37)
Mean score of work related stress (range, SD)	7. 09 (3–12, 2. 47)
Mean score of work place violence (range, SD)	5. 59 (0–27, 5. 31)
Mean score of self-esteem (range, SD)	30. 79 (16–38, 3. 64)
Mean score of life satisfaction (range, SD)	14. 20 (4–20, 4. 18)
Mean score of depression (range, SD)	14. 56 (0–50, 10. 94)

The mean scores of the continuous variables used in the pathway analysis are presented in Table 1 along with the ranges and standard deviations. The mean score of controlling by husband was 3, self-esteem was 31 and life satisfaction was 14. While, the mean scores of WPV, work related stress and depression were 6, 7 and 15 respectively.

Table 2 presents a comparison of background characteristics and life experiences of the women by the presence of depressive symptoms. Women with depressive symptoms were significantly older, and had more children, less ability to mobilize resources in emergency, lower self-esteem, lower life satisfaction, disability and high work stress. They were also more highly controlled by husbands, and a higher proportion of them reported their husband having extra marital sex. A higher proportion of women with depressive symptoms also reported IPV and WPV compared to the women who did not have any symptoms of depression.

**Table 2:** Socio-demographic characteristics and life experiences of female garment workers by presence of depressive symptomatology, n = 800.

Characteristics	% / mean	% / mean	*p* value
	Depressive symptoms		
*Worker characteristics*	*Yes*	*No*	
	*N = 318*	*N = 482*	
Age (in years) (%)			
15–19	3. 77	5. 39	0. 004
20–24	25. 79	33. 40	
25–29	32. 08	34. 44	
≥30	38. 36	26. 76	
Level of education (%)			
No education	22. 64	16. 60	0. 057
1–5 years of education	32. 39	38. 59	
≥ 6 years of education	44. 97	44. 81	
Age at marriage (categories) (%)			
< 15 years	37. 11	29. 05	0. 002
15–19 years	46. 54	59. 13	
> 19 years	16. 35	11. 83	
No. of children alive (categories) (%)			
No child	14. 78	17. 63	0. 015
One child	40. 57	47. 93	
Two or more child	44. 65	34. 44	
Member of NGO (%)	50. 34	49. 66	0. 004
Average current earning per month (BDT)	8511. 82	8500. 93	0. 9114
Income			
Tertile I (Lower income)	45. 28	45. 02	0. 824
Tertile II (Moderate income)	27. 04	25. 52	
Tertile III (High income)	27. 67	29. 46	
Ability to mobilize resources in emergency (%)			
Very difficult	70. 75	52. 49	0. 000
Somewhat difficult	18. 87	25. 10	
Fairly easy	7. 86	17. 63	
Very easy	2. 52	4. 77	
Self-esteem (mean)	28. 81	32. 09	0. 000
Life satisfaction (mean)	11. 62	15. 90	0. 000
Disability (mean)	22. 48	23. 40	0. 000
Controlling by husbands (mean)	3. 60	2. 71	0. 000
Husband’s extramarital sex (%)	11. 64	3. 94	0. 000
Work related stress (mean)	8. 43	6. 21	0. 000
Any IPV (%)	85. 53	58. 09	0. 000
Workplace violence (%)	84. 59	66. 18	0. 000

From the multivariate logistic regression analyses, the risk factors for depression indicated that women’s higher self-esteem (aOR 0. 84;95%CI 0. 79, 0. 90), higher life satisfaction (aOR 0. 86;95% CI 0. 81, 0. 91) and less disability (aOR, 0. 74;95% CI 0. 64, 0. 87) were significantly associated with a lower risk of depressive symptomatology (Table 3). While controlling behaviours from their husband (aOR, 1. 17;95% CI1. 04 1. 31), work related stress (aOR1. 21;95% CI 1. 10, 1. 33), any IPV (aOR 1. 71;95% CI 1. 06, 2. 75), and experiencing or witnessing WPV (aOR 1. 04;95% CI 1. 00, 1. 09) significantly increased the risk of depressive symptomatology (Table 3).

**Table 3:** Factors associated with depression among female garment workers: Results from multivariate logistic regression, n = 800.

Variables	aOR^a^	95% CI^b^	*p*-value
Age (in years)			
15–19 (ref)			
20–24	1. 04	0. 393–2. 772	0. 932
25–29	1. 38	0. 483–3. 913	0. 551
≥30	1. 70	0. 566–5. 105	0. 344
Education (in years)			
None (ref)			
1–5	0. 82	0. 481–1. 401	0. 469
≥6	1. 66	0. 955–2. 898	0. 072
Age at marriage			
< 15 years (ref)			
15–19 years	0. 67	0. 436–1. 026	0. 065
> 19 years	0. 97	0. 499–1. 861	0. 913
No. of children alive			
No child (ref)			
One child	0. 85	0. 459–1. 557	0. 591
Two or more child	0. 80	0. 377–1. 681	0. 547
NGO membership (ref no)	1. 22	0. 760–1. 965	0. 408
Income			
Tertile I (Lower income) (ref)			
Tertile II (Moderate income)	1. 40	0. 861–2. 212	0. 181
Tertile III (High income)	1. 03	0. 649–1. 628	0. 906
Ability to mobilize resources (indicator of SES)			
Very difficult (ref)			
Somewhat difficult	1. 08	0. 672–1. 729	0. 756
Fairly easy	0. 64	0. 341–1. 218	0. 176
Very easy	0. 77	0. 231–2. 587	0. 678
Husband’s extramarital sex	1. 31	0. 599–2. 877	0. 497
Controlling by husband	1. 17	1. 044–1. 313	0. 007
Self-esteem	0. 84	0. 787 - . 904	0. 000
Work related stress	1. 21	1. 103–1. 325	0. 000
Disability	0. 74	0. 636 - . 868	0. 000
Life satisfaction	0. 86	0. 808 - . 906	0. 000
Any IPV	1. 71	1. 063–2. 749	0. 027
Workplace violence	1. 04	1. 003–1. 086	0. 031

^a^Adjusted Odds Ratio

^b^95% Confidence Interval

**Fig 2:**
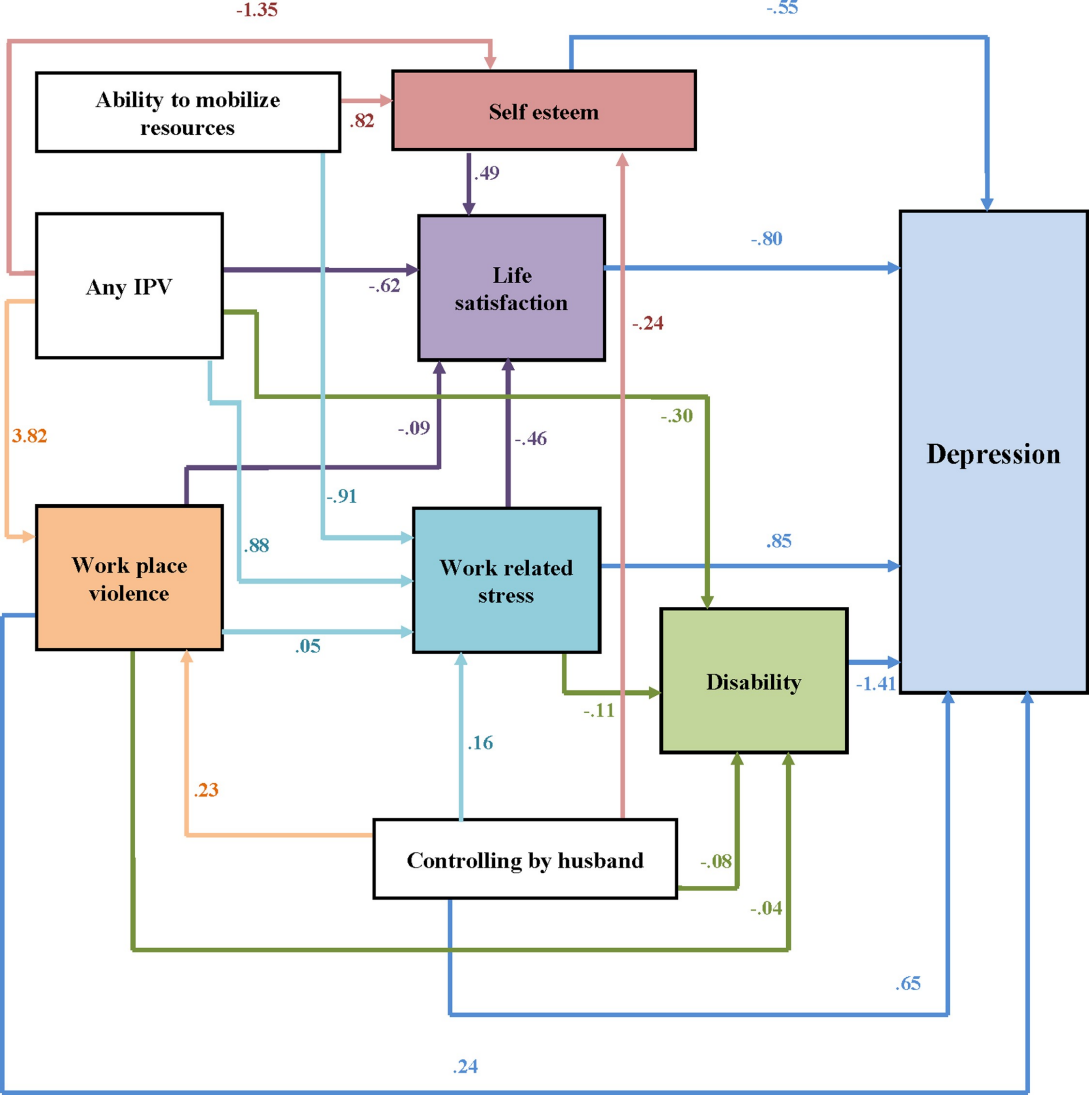
Path diagram: Depression among female garment workers.

The SEM shows the pathways (Fig 2) through which IPV and WPV leads to the development of depressive symptoms among female garment workers. Experience of any IPV did not have any direct effect on depression rather the paths between any IPV and depression were mediated through different life experiences. Life satisfaction (the higher IPV, the lower life satisfaction) mediated a path between IPV and depression (the lower life satisfaction, the higher depression). Self-esteem (the higher IPV, the lower self-esteem) mediated a second path between these variables and another path was mediated by both self-esteem and life satisfaction (The lower life satisfaction and self-esteem, the higher depression). WPV mediated the third path between IPV and depression (the higher IPV, the higher WPV and higher depression) and also by both WPV and work related stress. While the fourth path was mediated by work related stress (the higher IPV, the higher work related stress), which then mediate the path through life satisfaction (the higher work related stress, the lower life satisfaction) and disability (the higher work related stress, the higher disability). Worker’s disability also mediated a path between IPV and depression (the higher IPV, the higher disability and the higher depression).

WPV had a direct positive path to depression (whereby the higher WPV, the higher depression). The effect of WPV on depression is also mediated through various paths. The path is mediated by work related stress (the higher work related stress, the higher depression). Work related stress also mediated the paths through life satisfaction (the higher work related stress, the lower life satisfaction) and disability (the higher work related stress, the higher disability). Life satisfaction mediated a path between WPV and depression (the higher WPV, the lower life satisfaction and the higher depression). Another path is mediated by disability of the workers (the higher WPV, the higher disability and the higher depression).

A workers experience of controlling by her husband and her experience of WPV, work related stress and disability lead to depression. While there are additional paths between a worker’s self-esteem and work related stress were also highly affected by her ability to mobilize resources in emergency.

The standardized coefficients, standard errors, p-values, 95% confidence intervals, and variance of the disturbance and equation-level goodness of fit statistics, for the path model are presented in Table 4. The model fit was very good as root mean square error of approximation (RMSEA) = 0. 040, comparative fit index (CFI) = 0. 993 and Tucker-Lewis Index (TLI) = 0. 973.

**Table 4:** Path model: Depression among female garment workers, results from Structural Equation Model (SEM), n = 800.

Parameter	Coefficient	SE	P value	CI (95%)
Workplace violence → Depression	. 245	. 054	0. 000	0. 139, 0. 351
Life satisfaction → Depression	-. 800	. 085	0. 000	-0. 966, -0. 634
Controlling by husbands→ Depression	. 648	. 159	0. 000	0. 337, 0. 959
Self-esteem → Depression	-. 554	. 092	0. 000	-0. 735, -0. 373
Disability→ Depression	-1. 407	. 213	0. 000	-1. 824, -0. 991
Work related stress→ Depression	. 850	. 129	0. 000	0. 596, 1. 103
Any IPV → Workplace violence	3. 820	. 396	0. 000	3. 045, 4. 595
Controlling by husbands → Workplace violence	. 233	. 104	0. 025	0. 030, 0. 437
Workplace violence → Work related stress	. 048	. 015	0. 001	0. 019, 0. 078
Controlling by husbands → Work related stress	. 164	. 047	0. 000	0. 073, 0. 256
Any IPV → Work related stress	. 884	. 187	0. 000	0. 519, 1. 250
Ability to mobilize resources → Work related stress	-. 914	. 091	0. 000	-1. 093, -0. 735
Any IPV→ Self-esteem	-1. 349	. 276	0. 000	-1. 890, -0. 808
Controlling by husbands → Self-esteem	-. 244	. 073	0. 001	-0. 386, -0. 101
Ability to mobilize resources → Self-esteem	. 815	. 142	0. 000	0. 537, 1. 094
Any IPV→ Life satisfaction	-. 623	. 265	0. 019	-1. 142, -0. 104
Workplace violence→ Life satisfaction	-. 090	. 023	0. 000	-0. 135, -0. 046
Self-esteem→ Life satisfaction	. 489	. 034	0. 000	0. 422, 0. 556
Work related stress→ Life satisfaction	-. 459	. 051	0. 000	-0. 558, -0. 359
Any IPV →Disability	-. 303	. 108	0. 005	-0. 515, -0. 091
Workplace violence →Disability	-. 042	. 009	0. 000	-0. 060, -0. 024
Work related stress →Disability	-. 109	. 019	0. 000	-0. 147, -0. 072
Controlling by husbands →Disability	-. 080	. 027	0. 003	-0. 133, -0. 028
Disturbance variances	Estimates	SE	CI (95%)	
Depression	57. 605	2. 880	52. 228, 63. 537	
Work place violence	24. 488	1. 224	22. 202, 27. 009	
Work related stress	4. 938	0. 247	4. 477, 5. 447	
Self-esteem	11. 928	. 0596	10. 824,13. 156	
Disability	1. 602	0. 080	1. 452,1. 767	
Life satisfaction	10. 003	0. 500	9. 069, 11. 033	
Equation-level goodness of fit	R-squared			
Depression	0. 5058			
Work place violence	0. 1317			
Work related stress	0. 1912			
Self-esteem	0. 0979			
Disability	0. 1420			
Life satisfaction	0. 4202			
Overall	0. 3306			
Model fit information				
RMSEA	0. 040			
CFI	0. 993			
TLI	0. 973			

## Discussion

Our study extends the existing literature on IPV, WPV and depression by confirming the relationship between these experiences of violence (IPV and WPV) and depression. It also demonstrates that experiences of IPV and WPV increased depressive symptoms. Amongst female garment workers, IPV was common (69%), as was witnessing or experiencing WPV (74%), and clinically relevant depressive symptomology (40%).

In the adjusted regression model, experience of IPV and witnessing or experiencing of WPV was associated with increased depressive symptomology. These findings confirm previous research showing that these forms of VAW are independently associated with increased depressive symptomology amongst women [4,11,22]. Moreover, our findings also supports the inverse and direct relationship of work stress, life satisfaction, and self-esteem with depression [26,27,42].

Analysis using SEM showed that the association between IPV and depressive symptomology was mediated by a number of pathways. Thus, we find that experience of IPV impacts a woman’s experience of WPV and work related stress leading to the development of depressive symptomatology. These findings reinforce the fact that women’s experience of one form of violence increases the likelihood of other forms of abuse. The relationship between IPV and work related stress may be explained by job interference such as absenteeism, lower productivity and low concentration at work [43] and the fact that IPV affects work performance [11,43]. Experience of IPV compromises a woman’s ability to concentrate at work [44]and also leads to loss of working hours [45] increasing work related stress.

IPV also leads to depression through reduced self-esteem, life satisfaction and increased disability. These findings support and confirm the existing relationship between IPV and self-esteem [46], life satisfaction [47] and health [12].

WPV had direct and mediated pathways to depression. The direct pathway showed that increased WPV was associated with increased depressive symptoms. In addition, the relationship between WPV and depression was mediated by work related stress whereby WPV increases work related stress increasing depressive symptomatology. This is in line with the literature showing that workers who experience WPV are most likely to suffer from work related stress and are more likely to have physical and mental health problems [48]. Studies show that experience of WPV contributes to low productivity [20] and low job satisfaction [49], which in turn may lead to higher WPV and stress. Our findings extend the existing literature by showing that depression is heavily linked with life satisfaction and health [24,25,27] by showing the path and direction of these relationships.

Female garment workers’ experience of control by their husband leads to WPV and increased work related stress. The association between husband’s controlling behaviour and work related stress may be explained by a husband’s control over the worker’s earnings, which may exert extra strain on the worker [15]. All of these eventually contribute to depression. On top of these indirect links, controlling by husband also directly contributes to the development of depressive symptoms.

Ability to mobilize resources is fundamental to well-being [50] and it lowered the effect of work stress [51]. The SEM shows that ability to mobilise resources in an emergency is protective of depression, through mediated pathways showing it reduces work related stress, and in turn depression, and also increases life satisfaction reducing depression. The ability to mobilize resources may suggest that the worker has wider or stronger network/connections. Thus she may be relatively more empowered, capable of decision making and negotiating. All of which may make her less vulnerable to violence and depression.

Self-esteem, life satisfaction, work related stress and disability are the largest contributors to depressive symptoms among female garment workers. All these important predictors of depression were heavily affected by a worker’s experience of IPV and WPV.

The analysis showed that addressing both IPV and WPV is important for improving female garment worker’s mental health. The link between women’s ability to mobilize resources with self-esteem and work related stress indicates the need for socio-economic empowerment of women. All these may suggest that combined intervention to address IPV and women’s empowerment could be successful also in dealing with WPV and mental health. It is also important to change attitudes related to gender norms and gender-role beliefs that influence IPV. To achieve the full benefit of women’s economic empowerment addressing gender norms is important [52]. Previous literature suggests the relationship between negative gender attitudes and women’s experience of violence [53,54]. Thus, it is also important to work with men, to address gender norms as it is linked with controlling behaviour and IPV. Successful workplace based intervention to promote gender equity and address gender based violence [31,55] may be tested and replicated form other places. Mental health services must be an integral part of interventions to address IPV and WPV.

### Limitation

This study has some limitations. First, although path models are often stated as providing causal paths, any causality cannot be drawn from this analysis as data are cross-sectional. However, the reference periods for IPV and WPV reporting were longer than that of depression, suggesting a temporal ordering. Another limitation of the study is that prevalence of WPV cannot be estimated as we asked questions regarding experience of WPV and witnessing of such events at workplace in one item. It might be possible that the same event was witnessed and reported by several workers. However, this was the only possible way to gather information on WPV from workers in this setting, given that manager approval was required for the questionnaire. These findings are based on data from 800 female garment workers from eight selected factories and thus are not generalizable broadly in the garment sector or outside it. The women interviewed were from eight factories and whilst randomly selected, and so hopefully representative of the workers in their eligibility band per factory they may not represent all workers in their factories (including those with short periods of work there) nor those from other Dhaka factories.

## Conclusion

This study showed the complex relationship and the pathways through which experience of IPV and WPV leads to development of depressive symptoms among female garment workers. Women’s experience of IPV impacted on violence and stress at work resulting in development of depressive symptoms. This pathway analysis is expected to guide researchers, practitioners and policy makers to design and implement appropriate comprehensive intervention strategies to address IPV, workplace violence and to improve mental health of the female garment workers. This study clearly shows the stake of the garment industry in preventing violence and thus improving productivity. Addressing violence against women will benefit not only the individuals, families, the garment industry, but also contribute to development of the country as a whole.

## Supporting information

S1 Data Data to the manuscript. (DTA)
